# Our Hospitals and the National Relief Fund

**Published:** 1916-08-12

**Authors:** 


### The Hospital

The Workers' Newspaper of Administrative Medicine and Institutional Life, Administration, National Insurance and Health.

No. 1575, Vol. LX. SATURDAY, AUGUST 12, 1916. PRICE ONE PENNY.

### OUR HOSPITALS and the NATIONAL RELIEF FUND.

Like probably a majority of those who have contributed to build up the National Relief Fund, we have made a study of its methods, the grave faults it has committed, and the circumstances attending its grants in not a few cases, and we should welcome a new departure in the policy of those charged with the duty of the distribution of its funds. Two correspondents, Mr. J. Danvers Power in the *Times*, and Mr. A. D. Power in the *Daily Mail*, on successive days have suggested that a portion of the National Relief Fund should be placed at the disposal of King Edward's Hospital Fund for immediate distribution among our hospitals according to their needs. Mr. A. D. Power suggests £100,000 for distribution amongst the London hospitals, whilst Mr. Danvers Power, resting his plea upon precedence and for convincing reasons, advocates the claims of the hospitals of England and Wales for a share in the money of the National Relief Fund. He correctly insists that the successful work of King Edward's Hospital Fund for London, based as it is on sound principles, slowly adopted and amply justified by many years of practice and almost complete freedom from reasonable complaint, clearly points to its Distribution Committee as the best available authority in all matters connected with hospital finance either in London or in the provinces. If these really urgent suggestions meet with the favour which they merit, at least £250,000 will be handed by the National Relief Fund authorities to King Edward's Hospital Fund to meet the present pressing emergency of the voluntary hospitals in England and Wales.

We have had some distressing cases brought to out notice of civilian workers, men and women, whose earning power is stayed or seriously crippled owing to surgical troubles which it is claimed could be removed by prompt measures. Owing to the large number of beds which have been properly placed by the authorities of our voluntary hospitals at the disposal of the naval and military authorities, the waiting lists of some voluntary hospitals have grown to a length which demonstrates their emergency with cruel insistence. It seems to us that the statement of the position and claims of the hospitals by Mr. Harry Johnson on p. 451 demonstrates that there are none which will compare with those of our hospitals and the needs of the civil population which they represent. It is not our present purpose critically to examine the grants already made in all sorts of directions from the National Relief Fund. Let it suffice that the moneys in hand have come in response to an appeal the object of which was originally stated to be the relief of all hardship, whether arising directly from war casualties or through unemployment caused by the inevitable dislocation of trade. We agree with Mr. Danvers Power that the language might have been clearer, but we hold with him that the appeal it embraces certainly includes the present difficulties of voluntary hospitals, which come within it because they are in fact war contingencies.

For some months past we have been studying the position financially and generally of our voluntary hospitals. We have in preparation a number of facts which embrace the whole system of hospital relief and accommodation, which will have to be forthcoming after the war. The Commission of Investigation appointed by the Faculty of Insurance to inquire into the position of National Health Insurance, as we showed last week, must guard themselves against the wrong interpretation put upon some recent evidence which is claimed to vindicate the success of the Insurance Act so far as the hospitals are concerned. Party politicians' claims that the financial fears of hospital men have not been fulfilled are not justified, seeing that it is the voluntary hospitals, and not the Act, which have provided institutional treatment, the cost of which has not yet been repaid to the hospitals by the Government. The National Insurance scheme would have been in the Bankruptcy Court, long ago, had it not been for the generous admission of patients insured under the Act to treatment in the hospitals, which the system of medical relief set up by the Act failed to provide. We mention these facts to enable the Government and the authorities of the National Relief Fund to realise the claim of our voluntary hospitals to an immediate and substantial grant from a fund established "for the relief of all hardship, including that arising directly from war casualties," of which the exclusion of the civil population from immediate hospital treatment is probably the greatest and, in the interests of the people, the most urgent.

The claims of the voluntary hospitals are universally recognised. Few know them better or have expressed greater sympathy with them than several members of the present Ministry. This being so, seeing that the work done by the hospitals is of the first importance to the nation, we feel that the public interest demands spontaneous and immediate action on the part of His Majesty's Government and the authorities of the National Relief Fund.

